# An Induced Chromosomal Translocation in Soybean Disrupts a *KASI* Ortholog and Is Associated with a High-Sucrose and Low-Oil Seed Phenotype

**DOI:** 10.1534/g3.116.038596

**Published:** 2017-02-22

**Authors:** Austin A. Dobbels, Jean-Michel Michno, Benjamin W. Campbell, Kamaldeep S. Virdi, Adrian O. Stec, Gary J. Muehlbauer, Seth L. Naeve, Robert M. Stupar

**Affiliations:** * Department of Agronomy and Plant Genetics, University of Minnesota, St. Paul, Minnesota 55108; † Department of Plant Biology, University of Minnesota, St. Paul, Minnesota 55108

**Keywords:** soybean, fast neutron, translocation, sucrose, oil

## Abstract

Mutagenesis is a useful tool in many crop species to induce heritable genetic variability for trait improvement and gene discovery. In this study, forward screening of a soybean fast neutron (FN) mutant population identified an individual that produced seed with nearly twice the amount of sucrose (8.1% on dry matter basis) and less than half the amount of oil (8.5% on dry matter basis) as compared to wild type. Bulked segregant analysis (BSA), comparative genomic hybridization, and genome resequencing were used to associate the seed composition phenotype with a reciprocal translocation between chromosomes 8 and 13. In a backcross population, the translocation perfectly cosegregated with the seed composition phenotype and exhibited non-Mendelian segregation patterns. We hypothesize that the translocation is responsible for the altered seed composition by disrupting a β-ketoacyl-[acyl carrier protein] synthase 1 (*KASI*) ortholog. *KASI* is a core fatty acid synthesis enzyme that is involved in the conversion of sucrose into oil in developing seeds. This finding may lead to new research directions for developing soybean cultivars with modified carbohydrate and oil seed composition.

Seed composition traits are important targets for soybean [*Glycine max* (L.) Merr.] improvement as human and animal nutrition is largely dependent on the quality and quantity of seed constituents. Modern soybean cultivars contain ∼40% protein, 20% oil, 5% ash, and 35% carbohydrates on a dry matter basis. Of the carbohydrates, 11% are soluble, with sucrose contributing the largest portion (2.5–8.2% total dry seed weight) (Hymowitz *et al.* 1972; Openshaw and Hadley 1978; Liu 1997). The demand for high-quality soybeans has driven breeders to select for lines with higher protein, higher oil, and improved carbohydrate profiles (increased sucrose content and decreased raffinose and stachyose) (Taira 1990; Hagely *et al.* 2013).

Targeted improvement in soybean seed composition profiles is a goal for many breeding and genetic engineering programs (Hymowitz *et al.* 1972; Mazur *et al.* 1999; Herman *et al.* 2003; Fehr 2007). To increase the efficiency and precision of altering these traits, an understanding of the genes that regulate seed composition is needed. Many studies have been conducted to understand the metabolic pathways governing the accumulation of seed constituents; however, much less is known about the regulation of the partitioning between the various pathways (Ruuska 2002; Santos-Mendoza *et al.* 2008; Chen *et al.* 2009; Weselake *et al.* 2009; Hutcheon *et al.* 2010; Bates *et al.* 2013). A comprehensive understanding of the genes that regulate seed metabolism can inform and enable molecular breeding approaches for the development of novel seed composition traits. For example, the development of high-oleic acid soybean lines has been achieved through the identification and utilization of mutations in fatty acid desaturase genes (Pham *et al.* 2010; Haun *et al.* 2014). In order to facilitate future genetic gains, it is important to identify new genetic variation that can be utilized by breeders to improve seed composition.

Mutagenized populations, created via irradiation or chemical mutagenesis, serve as valuable tools for creating new phenotypic variation and studying gene function. Chemical mutagens, such as ethyl methanesulfonate or N-methyl-N-nitrosourea, have been effective at producing point mutations in soybean for studying gene function (Cooper *et al.* 2008; Dierking and Bilyeu 2009; Hudson 2012). Ultraviolet, X-ray, or FNs are irradiation mutagen sources that often result in large-scale structural changes on chromosomes (Li *et al.* 2016). Soybeans have been shown to be resilient to the genome aberrations induced by FNs, including large deletions, duplications, and translocations (Findley *et al.* 2011; Bolon *et al.* 2014; Stacey *et al.* 2016). In subsequent follow-up studies, mapping has been done to associate FN-induced structural variations with traits such as alterations in seed composition, short petioles, and gnarled trichomes (Bolon *et al.* 2011, 2014; Campbell *et al.* 2016).

The overall objective of this study was to identify the causal variant underlying a soybean mutant with altered seed sucrose and oil content. A soybean FN population (Bolon *et al.* 2011, 2014) was screened and a high-sucrose/low-oil mutant was identified. This mutant line was identified as having almost twice the amount of sucrose (8.1% on dry matter basis *vs.* a wild-type value of 4.7%) and less than half the amount of oil (8.5% on dry matter basis *vs.* a wild-type value of 19.6%) as compared to wild type, while maintaining average levels of protein content (38.2% on dry matter basis *vs.* a wild-type value of 39.7%). Subsequent mapping and genomic analyses revealed that the region responsible for this trait localizes to a FN-induced translocation that disrupted the coding region of an ortholog of *KASI*, a gene involved in regulating carbohydrate metabolism during seed filling.

## Materials and Methods

### Plant material and mutant screening

A FN mutant soybean population was developed at the University of Minnesota (Bolon *et al.* 2011, 2014) through the irradiation of soybean line “M92-220” (Orf and Denny 2004) and was the source for the mutant line used in this study. The mutant of interest was identified based on its seed phenotype, which consisted of a high-sucrose and low-oil seed composition. Plants within the lineage of this mutant have been given several names in previous publications and databases, including “FN0176450,” “FN0176450.xx.xx.xx.xx.06.M7,” “2012CM7F040P06,” and “R64C50.” In this paper, this mutant will herein be referred to as either “FN0176450” or “the high-sucrose/low-oil mutant.”

**Figure 1:**
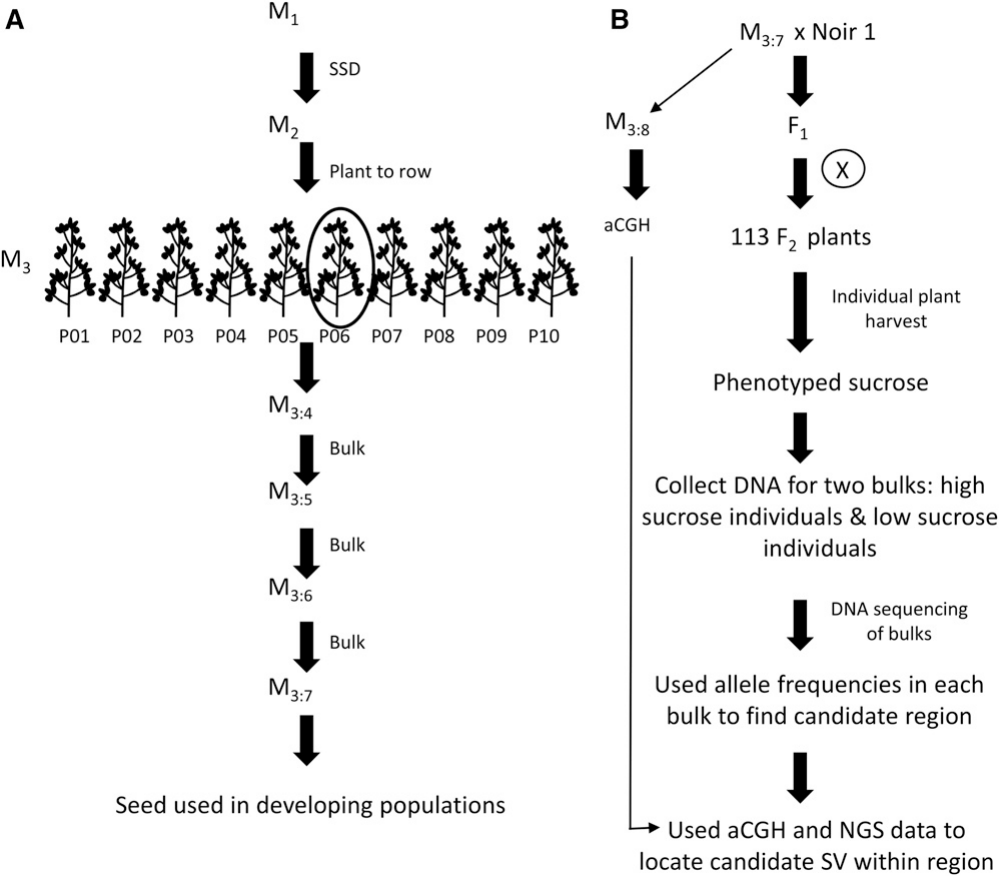
Flow chart of mutant line advancement and mapping. (A) Diagram displays how the mutants were advanced starting at the M_1_ generation. (B) A flow chart of the development of the F_2_ populations used for mapping and the steps taken to identify the structural variant associated with the phenotype of interest (high-sucrose/low-oil). aCGH, array comparative genomic hybridization; NGS, next-generation sequencing; SSD, single-seed-descent; SV, structural variation.

The high-sucrose/low-oil mutant was first identified from a mutant screen of ∼17,000 M_2_ individuals (Bolon *et al.* 2011, 2014). Nondestructive, whole-seed phenotyping was done on the population using Near Infrared (NIR) Spectroscopy at the University of Minnesota NIR Spectroscopy lab (St. Paul, MN) using a Perten DA7200 diode array instrument (Huddinge, Sweden) equipped with calibration equations developed by Perten in cooperation with the University of Minnesota. Lines of interest based on initial phenotypic screens were advanced, including FN0176450 (Figure 1A). More detailed descriptions of the FN population have been previously described (Bolon *et al.* 2011, 2014). Briefly, single M_2_ plants were grown in a grid pattern, the resulting M_3_ seed from each individual plant was planted in plant rows, and 10 individuals were harvested from each row creating families of 10 plants each. Subsequent generations were bulk-harvested until the M_3:7_ generation (Figure 1A).

Two separate populations were created with FN0176450, an outcross population for mapping the causal variant and a backcross population for validating the cosegregation of the variant with the phenotype. To generate the outcross population, the M_3:7_ generation of FN0176450 was crossed to cv. “Noir 1” subline Noir1-SGC-01 (herein called “Noir 1”) (McHale *et al.* 2012). The F_1_ seed was planted in a greenhouse, and the segregating F_2_ population was grown on 30-inch rows at St. Paul, MN during the summer of 2014. All F_2_ individuals were tagged, and fresh leaf tissue was collected and freeze-dried for later DNA extraction of select individuals. The plants were individually harvested and the F_2:3_ seeds were analyzed for sucrose and oil content. The backcross population was created by crossing FN0176450 to “M92-220” (Figure 1B). The F_1_ seed was planted in a greenhouse, and the BC_1_F_2_ population was grown on 30-inch rows at St. Paul, MN during the summer of 2015. All BC_1_F_2_ individuals were tagged, and fresh leaf tissue collected and freeze-dried for later DNA extraction of all individuals. At maturity, the BC_1_F_2_ plants were individually harvested and the BC_1_F_2:3_ seeds were analyzed for sucrose and oil content.

### Seed composition phenotyping

Seed composition profiles of the high-sucrose/low-oil mutant, the wild-type line (“M92-220”), and the progeny from both the outcross and backcross populations were assessed using multiple methods. Seed composition profiles of all samples in this study were determined using NIR spectroscopy of whole seeds (described above). NIR was considered sufficient for quantifying total protein and oil composition.

Chemical analysis to determine soluble carbohydrate (sucrose, raffinose, and stachyose) content of the wild-type and high-sucrose/low-oil mutant lines was done at the University of Missouri (UMO) Agricultural Experiment Station Chemical Laboratories (Columbia, MO) where gas–liquid chromatography (GLC) was used to quantify sugar content (Bhatti *et al.* 1970; Janauer and Englmaier 1978). Further chemical analysis of seed, including the high-sucrose/low-oil mutant “M92-220” and bulks of segregating BC_1_F_2_ plants, were analyzed at Eurofins Nutrition Analysis Center (Des Moines, IA). Sucrose content at Eurofins was analyzed with the Association of Official Agricultural Chemists (AOAC) 982.14 reference method using HPLC and an Evaporative Light Scattering Detector. Crude fat/oil concentration at Eurofins was analyzed using the American Oil Chemist’s Society (AOCS) method Ac 3–44, and protein concentration at Eurofins was measured using methods AOAC 992.15, AOAC 990.03, and AOCS Ba 4e−93. These same bulks were also analyzed at the University of Tennessee gas chromatography lab (Knoxville, TN) for fatty acid composition as described in Hyten *et al.* (2004).

Wet chemistry analysis of soybean seeds is costly and requires large quantities of seed. Therefore, the hundreds of plants that required a sucrose phenotype in the segregating populations were assessed using in-house phenotyping methods. An enzymatic assay was modified from the assay described by Teixeira *et al.* (2012). Herein, this method will be referred to as the “colorimetric” assay. The assay combines the action of invertase and glucose oxidase and is adapted to a 96-well ELISA plate, allowing for high-throughput and cost-effective analysis of sucrose content on a dry weight basis (Teixeira *et al.* 2012). First, 20 seeds from each sample were ground using a Hamilton Beach chamber coffee grinder. The samples were dried in an oven at 105° for 5 hr and soluble carbohydrates were extracted with 80% ethanol at 70° for 90 min. Invertase (invertase from baker’s yeast, SKU I4504, Sigma-Aldrich) was used to hydrolyze sucrose into glucose and fructose. A glucose assay kit (Glucose assay kit, SKU 120003400A, Eton Bioscience) was used to quantify the glucose in the solution. The solution in each well became pink based on the concentration of glucose in the sample, and this was quantified by analyzing the absorbance at 490 nm with a microplate reader (BioTek Synergy 2, BioTek Instruments, Winooski, VT) and comparing observed values to a standard reference curve of known sucrose concentrations. These standard solutions were prepared using sucrose analytical standard (Sigma-Aldrich, SKU 47289) and prepared at concentrations of 0, 0.05, 0.075, 0.10, 0.125, 0.15, 0.175, 0.2, 0.225, 0.25, 0.275, and 0.30 g of sucrose/100 ml.

### Array comparative genomic hybridization (aCGH) to detect structural variants

Array comparative genomic hybridization (aCGH) is a microarray-based approach that can be used to identify structural variants, as previously described (Bolon *et al.* 2011, 2014; Haun *et al.* 2011). In this study, aCGH was performed on M_3:8_ plants that were direct descendants of the M_3:7_ mutants used in making crosses (see Figure 1B). The aCGH array (Agilent Technologies, Inc.) was utilized to compare signal intensities of mutant DNA and wild-type (“M92-220”) DNA hybridized to unique probes designed from the soybean reference genome sequence (version Glyma.Wm82.a2.v1; Song *et al.* 2016). The microarray was designed with one million probes tiled throughout the genome and spaced to enrich for genic regions. Labeling reactions with mutant (Cy3) and reference (Cy5) dye were performed with 500 ng DNA from both the FN0176450 mutant and wild-type “M92-220” leaf tissue. Labeled DNA was hybridized to the microarray for 66 hr at 67° according to the manufacturer’s instructions. Log_2_ ratios between the control and mutant hybridizations were calculated for each probe. Labeling, hybridization, washing, and data acquisition were all performed according to the manufacturer’s protocols. For each CGH run, the aberration algorithm (ADM-2) in the Agilent Genomic Workbench software (version 7.0.4.0) and the Agilent feature extraction (version 12.0.0.7) were used to extract raw data and call significant aberrations. Visual assessment of aCGH data was done using JMP Pro (version 12) software.

### BSA and next-generation sequencing (NGS)

BSA using whole-genome sequencing was performed on the F_2_ population (“Noir 1” × FN0176450) in a similar fashion as in previous experiments (Michelmore *et al.* 1991; Abe *et al.* 2012; Campbell *et al.* 2016). Two pools of bulks were created: a high-sucrose bulk and a low-sucrose bulk. These bulks consisted of 15 F_2_ individuals with the highest sucrose content in bulk 1 and 19 F_2_ individuals with the lowest sucrose content in bulk 2.

DNA from selected individuals was prepared from freeze-dried leaf tissue from the 2014 growing season. One DNeasy (QIAGEN DNeasy Plant Mini Kit) was used for each bulk using equal amounts of leaf tissue from each individual in the bulk. A total of 20 mg freeze-dried leaf tissue was used for each bulk and ground using a QIAGEN Tissue Lyser II. After DNA extraction, the bulks were sent to the University of Minnesota Genomics Center for Illumina NGS. Samples were sequenced on a HiSequation 2500 HO 100 paired-end run using v4 chemistry and run using Rapid chemistry.

The sequence data quality was checked with FastQC version 0.11.2 before and after sequence data alterations to ensure the data were of sufficient quality for downstream BSA applications. Cutadapt version 1.6 was used to trim adapter sequences and remove low-quality reads. A limit was set, at which reads were removed if adapter trimming resulted in reads smaller than 30 bp. Fastx toolkit version 0.0.14 was used to remove low-complexity sequences. Fastq quality trimmer in the fastxtoolkit was also used to remove reads with phred quality scores <20. In this pipeline, BWA mem (v. 0.7.10) was used for mapping, using the Wm82.a2.v1 reference genome sequence. A mismatch penalty was set in BWA to allow for approximately six high-quality mismatches per read. Variant calling was done using the Genome Analysis Tool Kit (GATK version 3.3 49) Unified Genotyper, calling only SNPs at Wm82.a2.v1 positions of the SoySNP50K platform (Song *et al.* 2013). Uninformative SNPs were removed, including low-quality points where the read depth was <10 and nonpolymorphic SNPs between the parents.

After uninformative data were removed, allele frequencies were calculated for each position in the two bulks. A python script (called “VCF_MAF”) was used to calculate the allele frequency of the alternate allele at each of the 50 K positions based on allele depth ratios at each position. This script was used in a previous experiment (Campbell *et al.* 2016) and is publicly available at “https://github.com/TomJKono/Misc_Utils.” To calculate the percentage of the FN0176450 parent allele in each of the bulks, reference allele frequency was used (1-alt allele frequency) if the mutant parent allele was the same as the reference (genotype Williams 82) allele. The alternate allele frequency was used if the mutant parent allele was the same as the alternate allele. The allele frequencies were plotted and visually analyzed for spreads in allele frequency between the two bulks, as previously demonstrated (Campbell *et al.* 2016). NGS was also performed on the FN0176450 line using the same sequencing pipeline as that used in the bulked samples.

### Identification and validation of a chromosomal translocation associated with the high-sucrose/low-oil phenotype

After mapping, Integrative Genomics Viewer Version 2.3 (Broad Institute) was used to analyze Illumina sequencing data of the high-sucrose/low-oil mutant line. The association between the reciprocal translocation and the altered sucrose and oil seed content phenotype was tested by phenotyping and genotyping 93 plants in the backcross BC_1_F_2_ population. Phenotyping was done using both NIR and the colorimetric assay. A scatterplot was made using the ggplot2 package in R statistical software (Vienna, Austria). Genotyping was performed using translocation-specific PCR primers (Supplemental Material, Table S1 in File S1) designed using sequencing data from FN0176450.

### Data availability

Sequencing data for FN0176450 and “M92-220” is available in the NCBI Sequence Read Archive as accession numbers SRX467183 and SRX82634, respectively. Sequencing data for the two bulked samples are publicly available in the NCBI Sequence Read Archive as accession numbers SRX2438554 and SRX2438555, respectively. The comparative genomic hybridization data for the three replications comparing FN0176450 and “M92-220” can be found as accession number GSE93411 in the National Center for Biotechnology Information Gene Expression Omnibus (http://www.ncbi.nlm.nih.gov/geo). 

## Results

### Identification and seed composition profiling of a high-sucrose/low-oil mutant

An initial screen of several thousand soybean FN plants by NIR (Bolon *et al.* 2011) identified plant FN0176450 as a seed composition mutant. This line was advanced for its high-sucrose and low-oil phenotype, along with other lines that were at least 2 SDs above or below the mean of the population for seed composition traits. The phenotype of this mutant was measured over 6 yr and consistently showed an elevated sucrose and reduced oil seed composition phenotype compared to the wild-type “M92-220” line. The protein and oil measurements were determined by NIR analysis, while the sucrose measurements were determined by a combination of NIR and GLC. Seed sucrose and oil levels were significantly different every year between FN0176450 and M92-220, while seed protein levels were not determined to be statistically different. These differences observed for sucrose and oil content over the 6 year period indicated a heritable change induced by the FN mutagen. FN0176450 also exhibited a visible phenotype in the seed, including a whiter coloration and a more wrinkled seed coat than wild-type seeds (Figure S1 in File S1).

Seed harvested from the 2015 field season were analyzed by the Eurofins Nutrition Analysis Center to validate and better quantify the seed composition differences between FN0176450 and the wild-type parent. Results from these analyses showed major differences between the mutant and wild-type lines for sucrose, oil, and fatty acids. On average, the high-sucrose/low-oil mutant seeds had a 72% increase in sucrose content compared to wild type on a total seed dry weight basis (from 4.7 to 8.1% sucrose dry weight). In addition, the high-sucrose/low-oil mutant had an average reduction of 57% in seed oil content compared to wild type on a total dry weight basis (from 19.6 to 8.5% oil dry weight). For the fatty acid portion of the seed, the high-sucrose/low-oil mutant exhibited a 25% decrease in oleic acid compared to wild type (from 28 to 21% oleic acid in total fat) and a 120% increase in linolenic acid compared to wild type (from 6.5 to 14.5% linolenic acid in total fat). There were no major differences in protein content, raffinose, stachyose, or other fatty acids (palmitic, stearic, and linoleic) between the mutant and wild-type lines.

### Sucrose and oil phenotype maps to a translocation between chromosomes 8 and 13

BSA, aCGH, and NGS were used in combination to map the high-sucrose/low-oil mutant (Figure 1). First, aCGH was conducted to locate putative structural variants throughout the genome. The analysis of the aCGH data revealed many putative homozygous deletions on chromosomes 6, 7, 8, 10, 14, 16, and 18, ranging from 38 kb to 4 Mb (Figure S2 in File S1).

**Figure 2:**
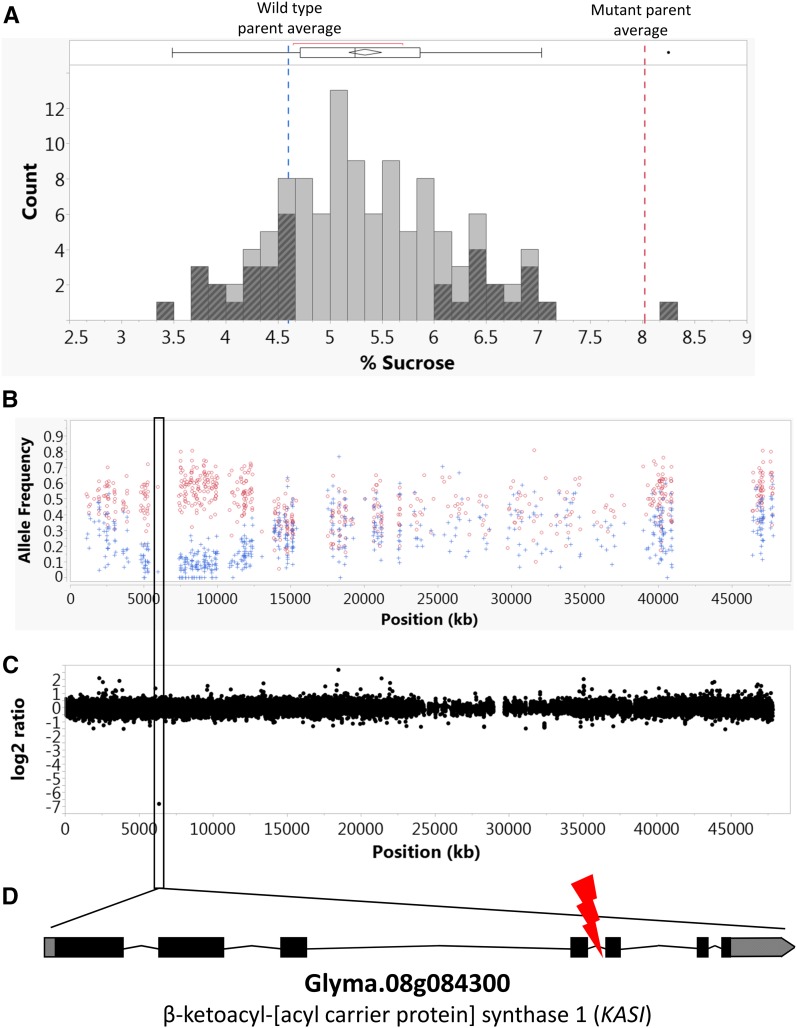
A flow chart of trait mapping. Bulked Segregant Analysis (BSA) was performed on an F_2_ outcross population to identify a structural variant in a β-ketoacyl-[acyl carrier protein] synthase 1 (KASI) ortholog associated with a high-sucrose/low-oil phenotype. (A) A histogram of sucrose content, as measured by the colorimetric assay, for the 113 individuals in the F_2_ population (“Noir 1” × FN0176450). Shaded histogram bars indicate those individuals that were selected for BSA and the vertical red and blue dashed lines indicate the parental phenotypes. Two bulks of DNA were formed (high-sucrose individuals and low-sucrose individuals) for whole-genome sequencing. (B) The allelic ratio for each single nucleotide polymorphism (SNP) along chromosome 8 in the high-sucrose (red data points) and low-sucrose bulks (blue data points). (C) The array comparative genomic hybridization (aCGH) graph displays the log_2_ ratio of the mutant genotype *vs.* the “M92-220” reference, where each dot represents a single aCGH probe. If the log_2_ ratio is <0, that indicates that the probe had a stronger signal intensity in wild type than in mutant, while a log_2_ ratio >0 would indicate a stronger signal intensity in mutant than wild type. A black box was drawn around a single probe with a low log_2_ ratio (log_2_ ratio = −6.5) highlighting that this probe was likely present in the wild-type line and absent in the high-sucrose/low-oil mutant line. (D) A structural variant between the fourth and fifth exon of Glyma.08g084300, indicated by the red lightning bolt.

Mapping was conducted to identify the structural variant associated with the high-sucrose/low-oil seed composition phenotype. FN0176450 was crossed to genotype “Noir 1” to generate a segregating F_2_ population for mapping. The distribution of seed sucrose content for the F_2_ population, as measured by a colorimetric assay, is displayed in Figure 2A, with shaded bars indicating the individuals selected for the high- and low-sucrose bulks for BSA. Across the genome, each bulk is expected to have approximately equal ratios of DNA from each parent. However, at SNPs surrounding the causative QTL, a spread in allele frequencies is expected between the high- and low-sucrose bulks. Visual analysis was done on all 20 chromosomes for this spread in allele frequency, and a 9 Mb interval on chromosome 8 between positions ∼3 and ∼12 Mb was found to be of interest due to its conspicuous spread in allele frequencies between the two bulked samples (Figure 2B). A closer look at the aCGH data in this interval revealed a single probe (Gm_CUST_P11209911) with a log_2_ ratio of −6.8 in FN0176450 at position 6,358,158 bp, between the fourth and fifth exon of a single gene model Glyma.08G084300 (Figure 2, C and D). Spurious data points can occur in aCGH analyses; however, this particular probe was consistently observed to indicate a deletion at this locus across three replicated aCGH hybridizations.

Analyses of the FN0176450 resequencing paired-end read data were subsequently used to infer the nature of the structural variation at this chromosome 8 position. A single base pair deletion was found within the sequence that matched the aCGH probe of interest. Furthermore, it was observed that reads flanking the 1 bp deletion were paired with reads mapping to chromosome 13. Moreover, the reads mapping to this chromosome 13 region were located near a 3 bp deletion. It was determined that numerous read pairs exhibited respective reads that mapped to chromosomes 8 and 13 at regions flanking the 1 and 3 bp deletions, indicating that a reciprocal translocation may have occurred between these regions. Subsequent PCR amplification between the chromosome 8 and 13 regions, and Sanger sequencing of the PCR products, confirmed the presence of the reciprocal translocation between the two chromosomes (Figure S3 and Table S1 in File S1).

The chromosomal translocation disrupts a gene (Glyma.08G084300) on chromosome 8 between the fourth and fifth exons (Figure 2D) and does not affect any predicted genes on chromosome 13. The closest *Arabidopsis* ortholog of Glyma.08G084300 is AT5G46290.1, which is annotated as *KASI* (Wu and Xue 2010). *KASI* is responsible for playing crucial roles in fatty acid synthesis (Figure S4 in File S1), chloroplast division, and embryo development (Wu and Xue 2010). Glyma.08G084300 has one close paralog in soybean, gene model Glyma.05g129600 (Schmutz *et al.* 2010; Song *et al.* 2016).

### Validation of chromosomal translocation association with high-sucrose/low-oil phenotype

The association of the reciprocal translocation between chromosomes 8 and 13 and the phenotype was validated by analyzing the seed composition profiles in a segregating backcross population developed by mating FN0176450 to M92-220. A total of 93 BC_1_F_2_ individuals were genotyped using four separate primer pair combinations, respectively designed to detect the wild-type chromosome 8, the wild-type chromosome 13, the 8–13 translocation junction, and the 13–8 translocation junction (Table S1 in File S1). The individuals were also phenotyped for sucrose content using the colorimetric assay and were assayed for protein, oil, fatty acids, and amino acids using NIR.

**Figure 3:**
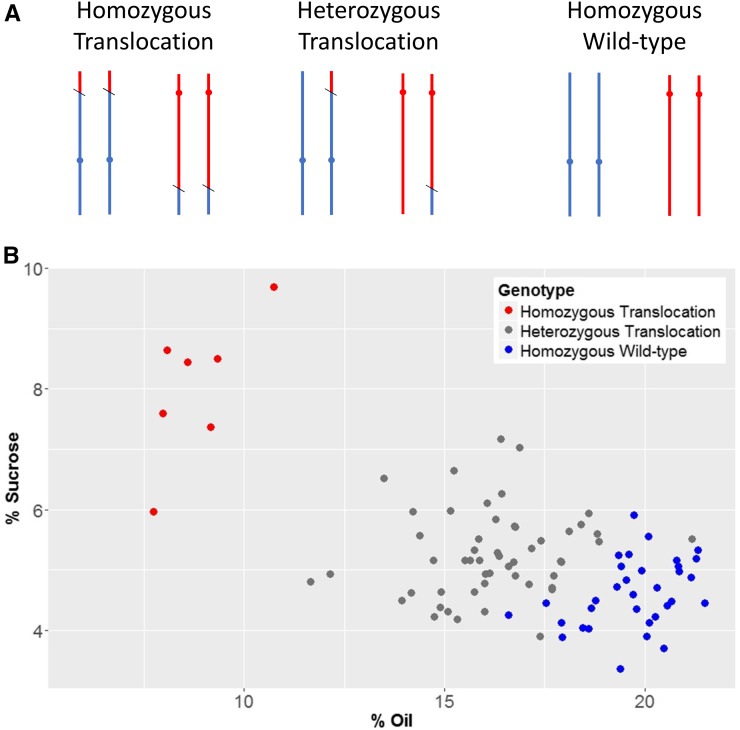
The association between the reciprocal translocation and seed sucrose and oil content. (A) Chromosome 8 (blue) and chromosome 13 (red) in homozygous translocation, heterozygous translocation, and homozygous wild-type states are shown. (B) The sucrose and oil seed composition in which each data point represents an individual in the BC_1_F_2_ population and colored by genotype class.

Three genotypic classes were identified in the population using the PCR assays, consisting of homozygous wild type, heterozygous translocation, and homozygous translocation (Figure 3A). Out of the 93 individuals genotyped, 33 were homozygous wild type, 53 heterozygous translocation, and seven homozygous translocation. Table 1 displays the average seed composition phenotypes of each of these three classes. There was a significant difference for both sucrose content (*P* < 0.0001) and oil content (*P* < 0.0001) for each of the three classes, with cosegregation of the translocation and the increased sucrose/decreased oil phenotype (Figure 3B). The seed phenotypes observed in the homozygous translocation BC_1_F_2_ individuals exhibited no significant difference compared to the mutant parent FN0176450 (*P* = 0.15). Similarly, the seed phenotypes observed in the homozygous wild-type BC_1_F_2_ individuals exhibited no significant difference compared to the wild-type parent M92-220 (*P* = 0.083). F_2:3_ plant rows of select BC_1_F_2_ individuals grown in 2016 showed the same results as the individual plant analysis in 2015. All of these results combined indicate that the mutant seed composition phenotype is fully explained by the translocated locus.

**Table 1:** Seed composition profiles of homozygous translocation, heterozygous translocation, and homozygous wild-type genotypes in the BC_1_F_2_ population

Genotype	Sucrose (%)	Oil (%)	Protein (%)	Raffinose (%)	Stachyose (%)	Palmitic Acid (%)	Stearic Acid (%)	Oleic Acid (%)	Linoleic Acid (%)	Linolenic Acid (%)
Homozygous translocation	8.02 a	8.80 c	43.20 ab	0.91 a	4.94 a	12.83 a	4.58 a	21.85 b	46.19 a	15.27 a
Heterozygous translocation	5.26 b	16.23 b	44.86 a	0.48 b	3.80 b	11.48 b	4.78 a	33.33 a	46.79 a	9.03 b
Homozygous wild type	4.61 c	19.71 a	42.81 b	0.37 c	3.13 c	10.93 c	4.68 a	35.09 a	47.73 a	7.44 c

The five fatty acids (palmitic, stearic, oleic, linoleic, and linolenic) are shown on a percent of total fatty acid basis and all other measurements are based on percent of seed, on a dry matter basis. Sucrose composition was analyzed using a colorimetric assay and all other measurements are based on near infrared spectroscopy. Means that do not share the same letter (a, b, c) are significantly different at *P* < 0.05.

## Discussion

### KASI as candidate gene for seed composition traits

This study utilized BSA, aCGH, and NGS to locate a FN-induced reciprocal translocation associated with increased sucrose and decreased seed oil content in soybean seeds. This association was validated in a more uniform genetic background through the creation and subsequent phenotyping and genotyping of a backcross population. The cosegregation between the translocation and the phenotype provides good evidence of the association. In addition, a single gene (Glyma.08G084300) was disrupted by the translocation event and is a clear candidate gene for this phenotype as it is an ortholog of *KASI* in *Arabidopsis*, which also exhibits a similar seed composition phenotype of reduced total oil content (Wu and Xue 2010).

The first functional characterization of *KASI* was performed in *Arabidopsis*. It was found that *KASI* is involved in fatty acid synthesis and chloroplast division and development (Wu and Xue 2010). In addition to *Arabidopsis*, work has been done in barley, rice, peanut, and tobacco to isolate and characterize *KASI* genes (Siggaard-Andersen *et al.* 1991; Chi *et al.* 2010; Domoney *et al.* 2013; Ding *et al.* 2015; Yang *et al.* 2016). Some of these studies suggested that silencing of *KASI* leads to decreased oil accumulation and altered fatty acid composition in seed, and impaired root cell elongation.

In addition to altered seed chemical profiles, the FN0176450 mutant line also showed a wrinkled seed phenotype (Figure S1 in File S1). This phenotype is similar to that seen in an *Arabidopsis* mutant of *wrinkled1* (*wri1*) (Focks and Benning 1998), which encodes an AP2/EREBP transcription factor that alters the transcription of genes related to the biosynthesis of seed storage compounds (Ruuska 2002). *Arabidopsis* mutants of *wri1* had an 80% reduction in seed oil content accompanied with increased sucrose and glucose content, indicating that *wri1* is involved in the regulation of carbohydrate metabolism (Focks and Benning 1998).

Mutants of *wri1* in *Arabidopsis* showed altered fatty acid composition profiles similar to that of the FN mutant presented here. The FN mutant in this study showed a 7% point reduction in oleic acid content (21% of the total fat, as compared to 28% in wild type). This is similar to the 10% point reduction shown in the *Arabidopsis wri1* mutant (13% of the total fat, as compared to 23% in wild type). Furthermore, the linolenic acid content was increased by 8% points in the FN mutant (14% of the total fat, as compared to 6% in wild type) and by 6% points in the *Arabidopsis wri1* mutant (16% of the total fat, as compared to 10% in wild type). As suggested by Focks and Benning (1998), this could be a result of reduced carbon flux into fatty acids. The similarity of the *wri1* and FN0176450 phenotypes suggests that disrupting the *KASI* ortholog in FN0176450 caused both the high-sucrose/low-oil phenotype as well as the wrinkled seed phenotype. Furthermore, it is worth noting that the disrupted soybean *KASI* ortholog in this study (Glyma.08G084300) has a paralogous copy (Glyma.05g129600), whereas *Arabidopsis* only appears to have one functional *KASI* gene. It is likely that a full knockout in *Arabidopsis* would have a strongly deleterious or lethal affect, and may explain why no known knockout line has been identified to date. In soybean, one might hypothesize that the paralogous copies have fully or nearly identical functions, but knockout of one copy reduces the metabolic flux of this function resulting in a viable but dramatically altered seed composition. This hypothesis is partially supported by the gene transcription of the two paralogous copies, which exhibit similar transcription levels across all tissues (Severin *et al.* 2010).

The silencing or knockdown of *wri1* leads to a reduction in seed oil content while the overexpression of *wri1* causes an accumulation of triacylglycerols in developing seeds of *Arabidopsis*, camelina, and maize (Focks and Benning 1998; Cernac and Benning 2004; Pouvreau *et al.* 2011; An and Suh 2015). In *Arabidopsis*, only a few genes involved in seed metabolism (45 of the >3500 genes) were differentially transcribed in the *wri1* mutant compared to wild type. *KASI* was among the few seed metabolism genes that exhibited differential transcription (Ruuska 2002), providing further evidence that *KASI* may be involved in the central metabolism of carbohydrates.

### Inheritance and genetics of the high-sucrose/low-oil phenotype

The inheritance patterns of the translocation in the BC_1_F_2_ population did not match Mendelian expectations, as assessed by the presence and absence of the four PCR assays among the 93 segregating individuals. First, proper Mendelian segregation would predict nine different marker presence/absence combinations among the four PCR assays. However, only three combinations were observed, matching those expected of homozygous wild-type, homozygous translocated, and heterozygous translocated individuals. These three classes are each expected to arise from the fertilization between balanced gametes, wherein each gamete either carries both wild-type chromosomes 8 and 13, or both reciprocally translocated chromosomes (8–13 and 13–8). The remaining six classes, which were not observed, would have been derived from unbalanced gametes, carrying one wild-type chromosome with a translocated chromosome segment. The lack of progeny from these six classes indicates that the duplication and deficiencies harbored by the meiotic cosegregation of a wild-type and translocated chromosome results in nonviable gametes.

Given that only balanced gametes were viable in this population, the predicted Mendelian ratio of the three observed genotypic classes would be (1) “homozygous wild type”: (2) “heterozygous translocation”: (1) “homozygous translocation.” While the ratio of homozygous wild type to heterozygous translocation approximated 1:2 (33:53 was observed), the seven homozygous translocated individuals were much lower than expected. This is likely the result of reduced germination of the mutant seed. In fact, the mutant parent line exhibited a germination rate of <50% across multiple years, supporting this hypothesis. It is possible that the fitness penalty of the homozygous translocation is attributable to the extreme high-sucrose/low-oil phenotype of the seed, and its effect on germination. This reduced fertility of the mutant is consistent with other studies that analyzed the fertility of lines harboring mutations in key fatty acid enzymes, including *KASI* (Mou *et al.* 2000; Wu and Xue 2010). Therefore, we speculate that the inviability of unbalanced gametes and the penalty on seed viability in the homozygous mutant *KASI*-like individuals explain the distorted segregation in this population.

Lastly, we hypothesize that the altered seed composition of the mutants is determined by the genetics of the seed rather than the genetics of the mother plant. This effect could be tested by phenotyping the segregation patterns of individual seeds derived from a single heterozygous mother plant. In this study, all phenotyping was performed on multiple seeds, following harvest from single plants. Heterozygous individuals in the backcross population had a phenotype (16.2% oil, 5.2% sucrose) that was intermediate compared to the wild-type segregants (19.7% oil, 4.6% sucrose) and the homozygous mutant segregants (8.8% oil, 8.0% sucrose). However, these data do not reveal whether the individual seeds from the heterozygous plant were essentially monomorphic or polymorphic for this phenotype. Assuming that unbalanced gametes are not viable, we would expect that the individual seeds from a heterozygous plant would segregate 1:2:1 for wild-type, intermediate (heterozygous), and mutant seed composition phenotypes. The phenotyping of multiple seeds together thus reveals an intermediate phenotype. While phenotype data from single seeds may exhibit high experimental variability, it may also reveal segregation within a single heterozygous plant, thus confirming the hypothesis that this mutant phenotype is controlled by the genetics of the seed *per se*.

### Conclusions

This study provides a candidate gene for further investigation on the regulation of carbohydrate metabolism in soybean seeds. It will be interesting to test alternative alleles of this gene, including nonknockout alleles and those with altered amino acid sequences, to identify different phenotypic outcomes. To our knowledge, there have not been any characterized mutants of this gene in soybean and further understanding of its role in regulating the accumulation of seed storage compounds will provide new methods for altering (and improving) the seed composition profile of elite soybean cultivars for human and animal nutrition and other end uses.

## Supplementary Material

Supplemental material is available online at www.g3journal.org/lookup/suppl/doi:10.1534/g3.116.038596/-/DC1.
