# Supplementary material for: An Induced Chromosomal Translocation in Soybean Disrupts a KASI Ortholog and Is Associated with a High-Sucrose and Low-Oil Seed Phenotype
Source: G3 (Bethesda). 2017 Feb 22;7(4):1215–23. doi: 10.1534/g3.116.038596 (PMC5386870; doi:10.1534/g3.116.038596)
Supplement: Supplementary file 1 [file 1215file001.doc]

**An induced chromosomal translocation in soybean disrupts a KASI ortholog and is associated with a high sucrose and low oil seed phenotype**

Austin A. Dobbels^*^, Jean-Michel Michno^*^, Benjamin W. Campbell^*^, Kamaldeep S. Virdi^*^, Adrian O. Stec^*^, Gary J. Muehlbauer^*^,^§^, Seth L. Naeve^*^, Robert M. Stupar^*,1^

^*^ Department of Agronomy and Plant Genetics, University of Minnesota, St. Paul, MN 55108

^§^ Department of Plant Biology, University of Minnesota, St. Paul, MN 55108

^1^Author for correspondence:

Robert M. Stupar

University of Minnesota

1991 Upper Buford Circle

411 Borlaug Hall

St. Paul, MN 55108-6026

Office: 612-625-5769

Fax: 612-625-1268

Email: stup0004@umn.edu


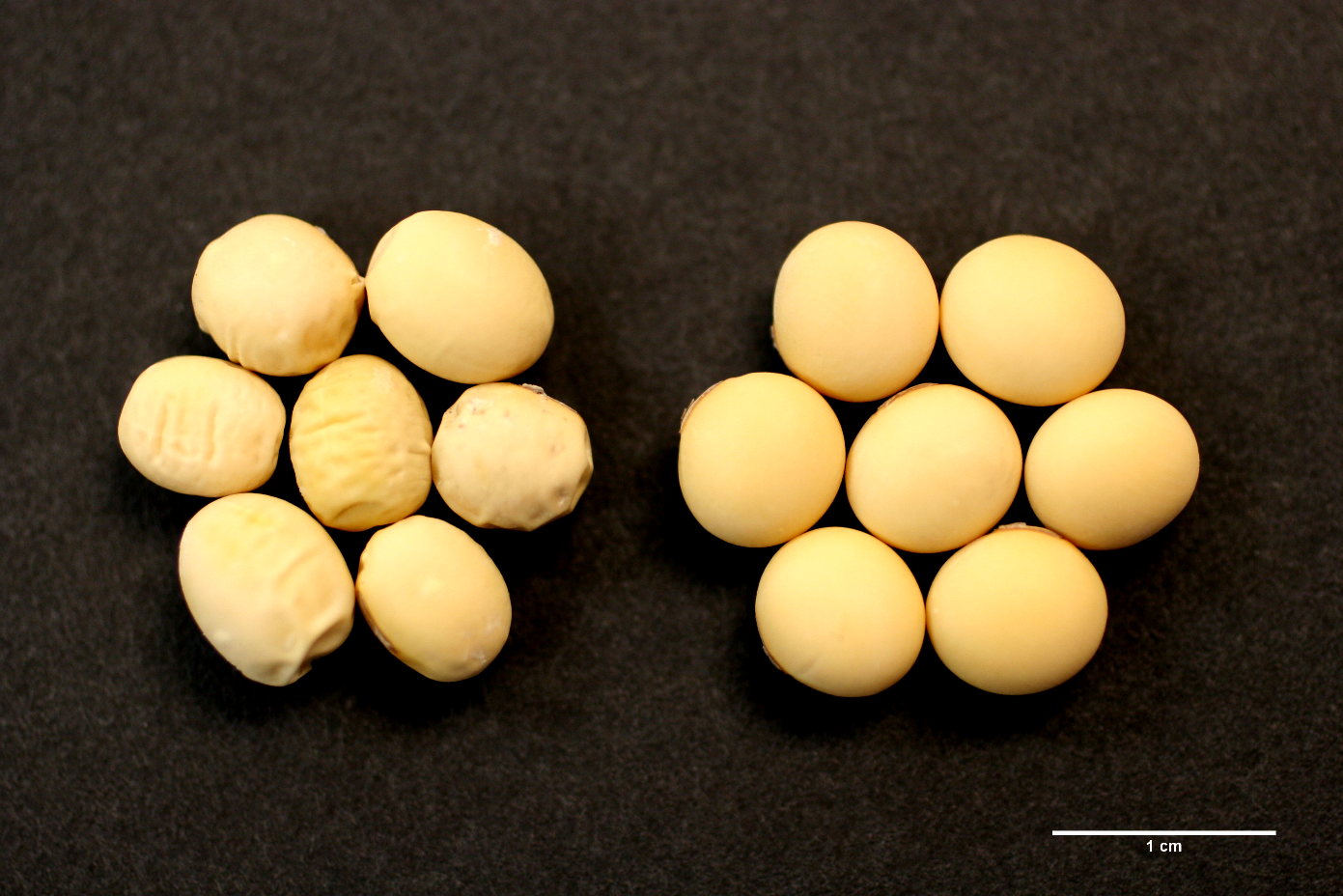


**Figure S1** High-sucrose/low-oil mutant and wild-type (‘M92-220’) show differences in seed size, shape, and color. The mutant seeds (left) appear to be slightly wrinkled, smaller, and lighter in color compared to wild type seeds (right).


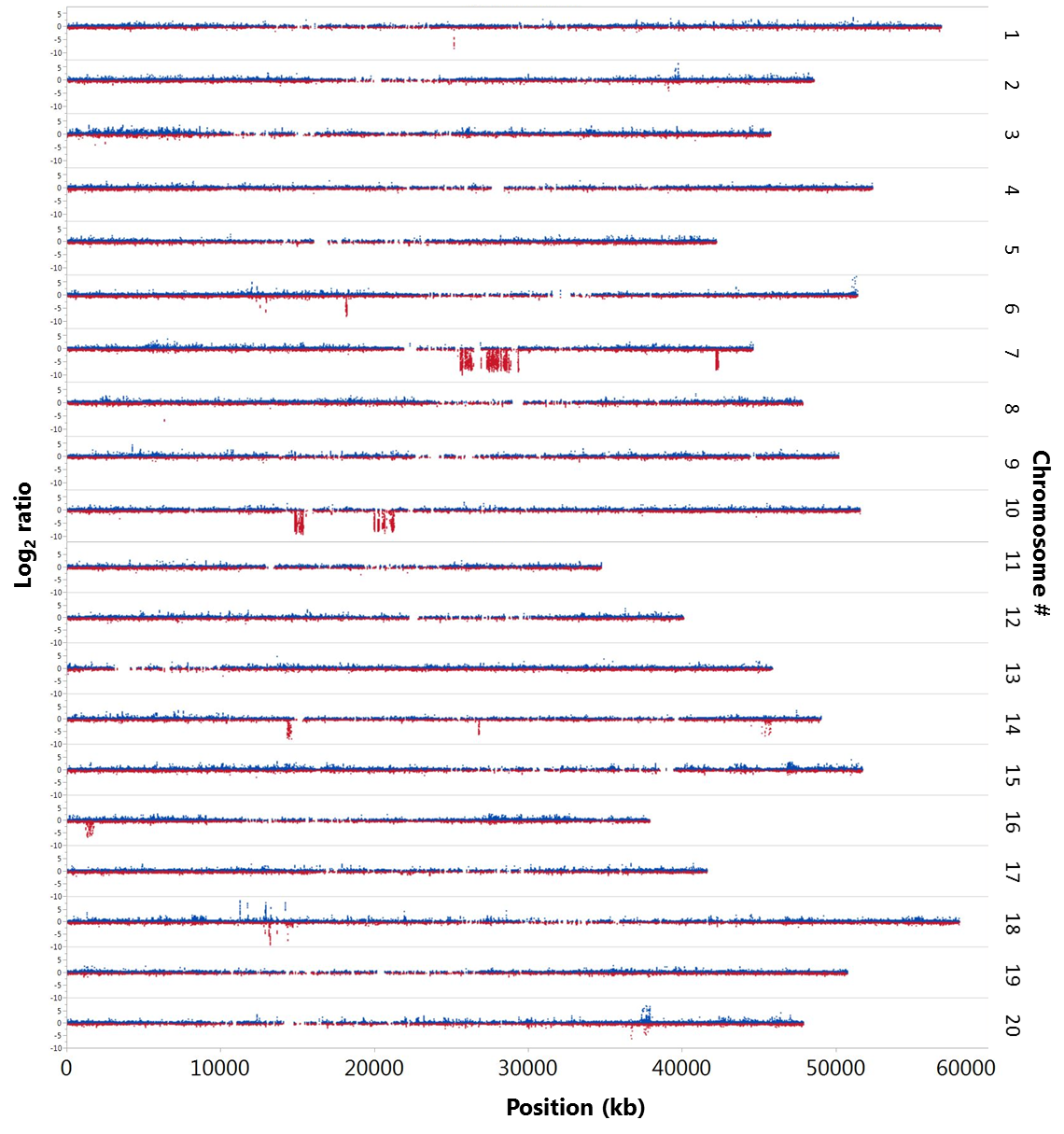


**Figure S2** Array-CGH data plotted for all 20 chromosomes in mutant line FN0176450. Graphed is the log2 ratio of the mutant genotype vs. the M92-220-Long reference where each dot represents a single aCGH probe. A log2ratio below 0 (colored red) indicates that the probe had a stronger signal intensity in wild-type than in mutant, while a log2 ratio above 0 (colored blue) would indicate a stronger signal intensity in mutant than wild-type.


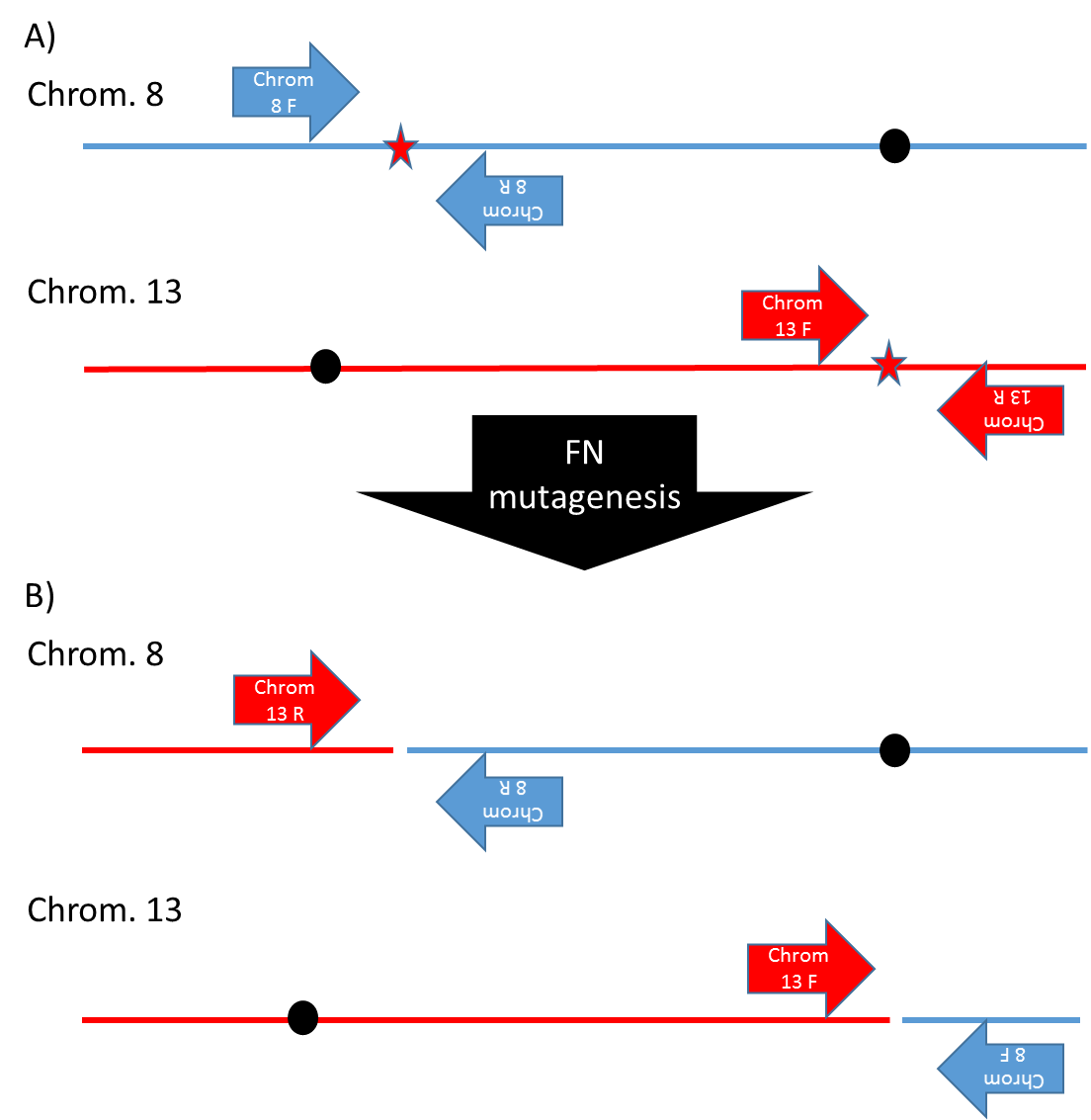


**Figure S3** Chromosome 8 and 13 reciprocal translocation diagram. Panel A depicts wild-type chromosome 8 (blue) and chromosome 13 (red). The red stars indicate the chromosomal breakpoint locations of the translocation. The red and blue arrows indicate the position and direction of the four primers developed to identify the translocation. PCR primers were named according to the chromosome they would amplify (Chromosome 8 or 13) and whether they were forward or reverse primers. Panel B shows the FN induced reciprocal translocation between chromosomes 8 and 13, and the figure shows the altered pairings of PCR primers as a result of the translocation. In addition, the black circles in both panels (A and B) indicate centromeres.

**
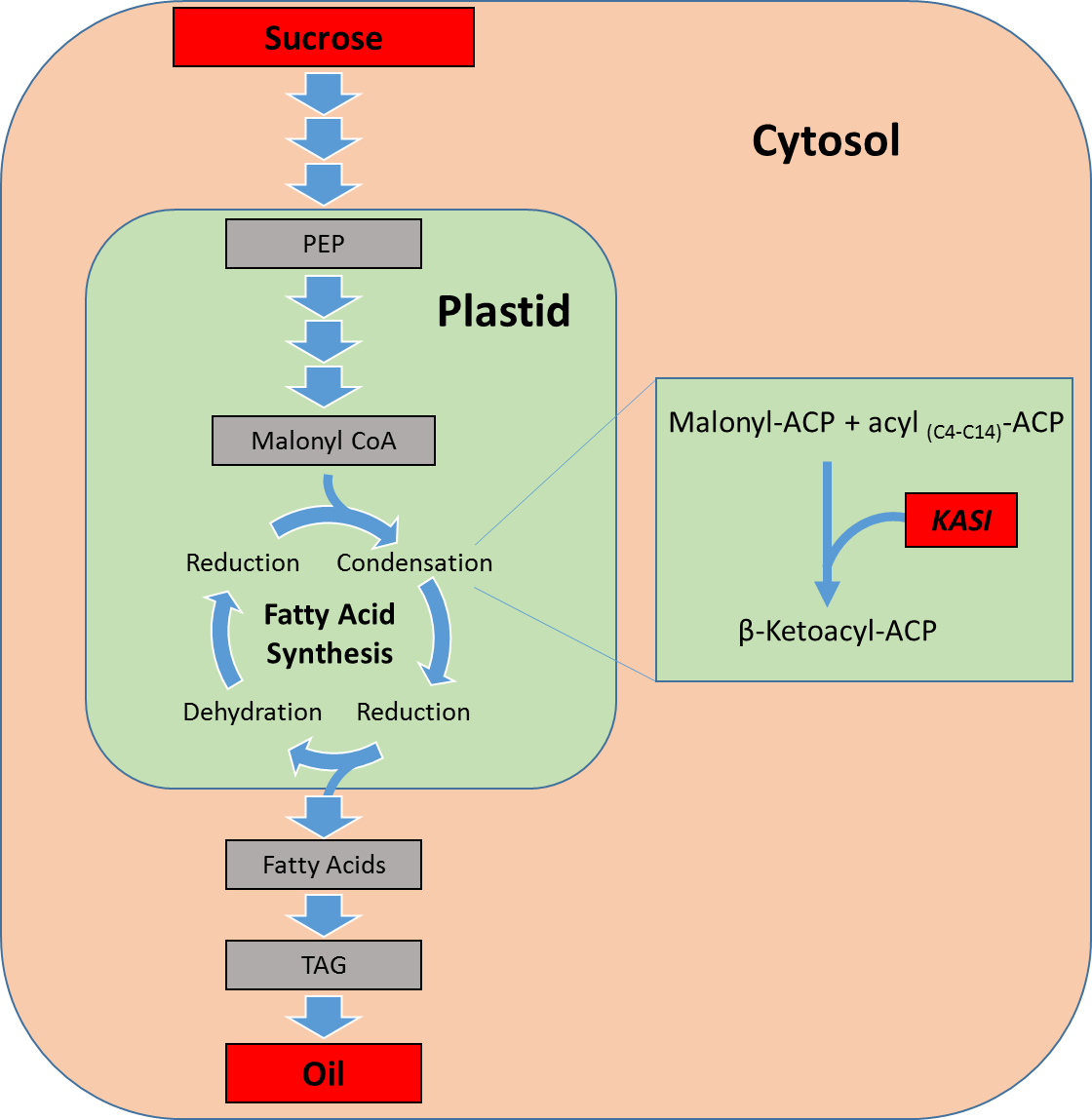
**

**Figure S4** Outline of sucrose to oil pathway in developing soybean seeds. This figure highlights the major enzymes and reactions in the pathway with some intermediates, enzymes, and transporters omitted for simplicity. Sucrose is transported into the cytosol via a sucrose transporter (not shown), and is metabolized into fatty acids through a series of biosynthetic pathways (Ruuska et al., 2002). The gene being disrupted in this study encodes KASI which is involved in the condensation stage of fatty acid synthesis and the elongation from C4 to C16. Abbreviated is: KASI, ketoacyl-synthase 1; PEP, phosphoenolpyruvate; ACP, acyl carrier protein; CoA, coenzyme A; TAG, triacylglyceride.

**Table S1** PCR primers used in detecting the reciprocal translocation. Displayed are the forward and reverse primers used to assay the chromosome 8 and 13 wild-type junctions and chromosome 8 and 13 reciprocal translocation junctions. Included are the primer names, primer sequences, and expected band sizes for each of these primer pairs in wild type (WT), mutant (Mut), and heterozygous (Het) individuals.

| **PCR Reactions** | | **Expected band size (bp)** | | |
| --- | --- | --- | --- | --- |
| **Forward primer ID:**  **Forward primer sequence** | **Reverse primer ID:**  **Reverse primer sequence** | **WT** | **Mut** | **Het** |
| P06_Chrom13_R1:  ACATCACTTGATGACTCCAGCA | P06_Chrom08_R1:  AAGCAATTGAGTCCACATGGCTA | - | 814 | 814 |
| P06_Chrom13_F1:  ATGAACTTGGCACCTCTCCC | P06_Chrom08_F1:  ACTCTTGCTGGAGACTTGGC | - | 489 | 489 |
| P06_Chrom13_F2:  TGGGCTTGAATGGTGACTCC | P06_Chrom13_R2:  ATCCTACAAACCAATCCGTGAA | 805 | - | 805 |
| P06_Chrom08_F3:  GGTTGTCAAGCCATCTAC | P06_Chrom08_R3:  TTTGCGAGCAATCTTTATG | 1990 | - | 1990 |
